# What containment strategy leads us through the pandemic crisis? An empirical analysis of the measures against the COVID-19 pandemic

**DOI:** 10.1371/journal.pone.0253237

**Published:** 2021-06-21

**Authors:** Daniel Kaimann, Ilka Tanneberg

**Affiliations:** Department of Management, Paderborn University, Paderborn, Germany; Italian National Research Council (CNR), ITALY

## Abstract

Since January 2020, the COVID-19 outbreak has been progressing at a rapid pace. To keep the pandemic at bay, countries have implemented various measures to interrupt the transmission of the virus from person to person and prevent an overload of their health systems. We analyze the impact of these measures implemented against the COVID-19 pandemic by using a sample of 68 countries, Puerto Rico and the 50 federal states of the United States of America, four federal states of Australia, and eight federal states of Canada, involving 6,941 daily observations. We show that measures are essential for containing the spread of the COVID-19 pandemic. After controlling for daily COVID-19 tests, we find evidence to suggest that school closures, shut-downs of non-essential business, mass gathering bans, travel restrictions in and out of risk areas, national border closures and/or complete entry bans, and nationwide curfews decrease the growth rate of the coronavirus and thus the peak of daily confirmed cases. We also find evidence to suggest that combinations of these measures decrease the daily growth rate at a level outweighing that of individual measures. Consequently, and despite extensive vaccinations, we contend that the implemented measures help contain the spread of the COVID-19 pandemic and ease the overstressed capacity of the healthcare systems.

## Introduction

The first reported and confirmed case of the novel COVID-19 pneumonia COVID-19 and its causative virus, SARS-CoV-2, was reported in Wuhan, China, on December 31, 2019 [1]. As of May 24, 2020, there have been 5,418,000 total confirmed cases of SARS-CoV-2 infections worldwide in 188 countries, including 344,742 deaths [2], and the outbreak is continuing at exponential growth [3]. At the beginning of January, first studies have suggested the likelihood of travel-related risks regarding the regional and global spread of the coronavirus [4]. Bogoch et al. [5] have stated that "the current outbreak in Wuhan, China serves as a reminder of how rapidly novel pathogens can appear and spread with potentially serious global consequences. Although it is unclear what the current burden of disease is or the potential for human-to-human transmission, major Asian hubs are the most probable sites of exportation should this epidemic continue […]".

Consequently, to keep the pandemic at bay, measures that limit population mobility and within-population contact rates should be considered in affected areas [4]. These include school closures, bans of gatherings, work-from-home covenants, and the most draconian measure, curfews, and total system shut-downs. This concept is also known as "flattening the curve", which involves decreasing and delaying an epidemic’s peak to avoid overstressing the capacity of healthcare systems [6]. During the ongoing pandemic with its persistent increases of new coronavirus infections, highly recurrent updates, even daily, on clinical outcomes and laboratory test results are crucially important for controlling public health planning both domestically and internationally [7, 8].

Since the outbreak of the SARS-CoV-2 pandemic, countries have implemented various measures to interrupt the transmission of the virus from person to person and prevent an overload of the health system. To name a few, India, China, France, Italy, New Zealand, Poland, Denmark, Austria, Germany, Spain, and the UK have established restrictive regulations on travel and private movement, placing a third of the global population in a coronavirus quarantine [9]. The implemented measures differ in their extent of restrictions for public and economic life, varying from school and business closures, travel entry restrictions, bans of gatherings, to national quarantines. For example, since March 17, 2020, France has been on a total lockdown, with all non-essential gatherings and excursions from home banned [10].

We aim to analyze the impact of such measures and combinations of these measures against the proliferation of the COVID-19 disease. Accordingly, we aim to address the following specific research questions:

1. *How do the different types of measures affect the containment of the spread of the COVID-19 pandemic*?
2. *How do different combinations of measures help against the proliferation of the SARS-CoV-2 virus*?

## Data

To analyze the impact of implemented measures against the COVID-19 pandemic, we use a sample of 68 countries, Puerto Rico and the 50 federal states of the United States of America, four federal states of Australia, and eight federal states of Canada, involving 6,941 daily observations between January 22 and May 24, 2020. Each country observation starts from the first confirmed case and ends either on May 24 or when one measure was first lifted. A list of the countries and states with their observed periods can be found in Table 1. This data has been obtained from the John Hopkins Coronavirus Resource Center [11], which combine data sources from the World Health Organization, the Centers for Disease Control and Prevention, the European Centre for Disease Prevention and Control, 1point3acres, Worldometers.info, Breaking News On, state and national government health departments, and local media reports.

**Table 1:** Countries and states and their observed periods.

Country	State	First observation	Last observation	Country	State	First observation	Last observation
Albania		2020-03-09	2020-04-19	Slovenia		2020-03-05	2020-05-03
Argentina		2020-03-18	2020-05-10	Sri Lanka		2020-02-18	2020-03-24
Armenia		2020-03-03	2020-05-03	Switzerland		2020-02-26	2020-04-26
Australia	New South Wales	2020-03-12	2020-05-14	Thailand		2020-01-22	2020-05-16
Australia	Queensland	2020-04-08	2020-05-10	Turkey		2020-03-19	2020-05-24
Australia	South Australia	2020-03-18	2020-05-14	United Kingdom		2020-02-27	2020-05-24
Australia	Victoria	2020-03-24	2020-04-10	Ukraine		2020-03-04	2020-05-10
Austria		2020-02-29	2020-04-13	United Arab Emirates		2020-01-29	2020-05-24
Bangladesh		2020-03-08	2020-05-09	Uruguay		2020-03-23	2020-05-23
Belgium		2020-03-01	2020-05-10	US	Puerto Rico	2020-03-15	2020-03-29
Bolivia		2020-03-15	2020-05-24	US	Alaska	2020-03-11	2020-04-24
Bosnia and Herzegovina		2020-03-09	2020-04-30	US	Alabama	2020-03-14	2020-04-29
Bulgaria		2020-03-09	2020-05-24	US	Arkansas	2020-03-12	2020-05-03
Canada	Alberta	2020-03-08	2020-05-13	US	Arizona	2020-03-02	2020-05-03
Canada	British Columbia	2020-03-20	2020-05-13	US	California	2020-03-06	2020-05-13
Canada	Manitoba	2020-03-26	2020-05-03	US	Colorado	2020-03-09	2020-04-30
Canada	New Brunswick	2020-04-01	2020-05-07	US	Connecticut	2020-03-08	2020-05-19
Canada	Newfoundland and Labrador	2020-03-28	2020-05-10	US	Delaware	2020-03-11	2020-05-07
Canada	Nova Scotia	2020-03-17	2020-04-30	US	Florida	2020-03-05	2020-05-24
Canada	Ontario	2020-02-05	2020-05-18	US	Georgia	2020-03-07	2020-05-24
Canada	Quebec	2020-03-17	2020-05-10	US	Hawai	2020-03-09	2020-05-06
Chile		2020-03-27	2020-05-24	US	Iowa	2020-03-10	2020-05-24
Colombia		2020-03-07	2020-05-24	US	Idaho	2020-03-12	2020-04-30
Cuba		2020-03-23	2020-05-24	US	Illinois	2020-03-04	2020-05-24
Cyprus		2020-03-29	2020-05-03	US	Indiana	2020-03-07	2020-05-03
Czech Republic		2020-03-02	2020-04-30	US	Kansas	2020-03-10	2020-05-03
Denmark		2020-02-27	2020-04-14	US	Kentucky	2020-03-07	2020-04-19
Denmark	Faroe Islands	2020-03-05	2020-05-02	US	Louisiana	2020-03-10	2020-05-14
El Salvador		2020-04-05	2020-05-24	US	Massachusetts	2020-02-03	2020-05-17
Estonia		2020-02-27	2020-05-10	US	Maryland	2020-03-07	2020-05-14
Ethiopia		2020-03-30	2020-05-24	US	Maine	2020-03-11	2020-05-10
Fiji		2020-03-19	2020-05-24	US	Michigan	2020-03-11	2020-05-06
Finland		2020-02-28	2020-05-12	US	Minnesota	2020-03-07	2020-04-26
France		2020-02-25	2020-03-15	US	Missouri	2020-03-12	2020-05-04
Gibraltar		2020-03-13	2020-04-30	US	Mississippi	2020-03-13	2020-04-26
Hungary		2020-03-06	2020-04-29	US	Montana	2020-03-11	2020-04-26
Iceland		2020-02-28	2020-05-03	US	North Carolina	2020-03-04	2020-05-07
Iran		2020-04-09	2020-04-19	US	North Dakota	2020-03-11	2020-05-14
Ireland		2020-03-19	2020-05-17	US	Nebraska	2020-03-08	2020-05-24
Israel		2020-02-26	2020-04-19	US	New Hampshire	2020-03-05	2020-05-02
Italy		2020-02-25	2020-05-03	US	New Jersey	2020-03-07	2020-05-17
Kazakhstan		2020-03-14	2020-05-03	US	New Mexico	2020-03-11	2020-05-15
Kuwait		2020-05-14	2020-05-24	US	Nevada	2020-03-09	2020-05-08
Latvia		2020-03-02	2020-05-14	US	New York	2020-03-06	2020-05-15
Liechtenstein		2020-03-05	2020-04-29	US	Ohio	2020-03-11	2020-05-03
Lithuania		2020-02-29	2020-04-27	US	Oklahoma	2020-03-08	2020-04-24
Luxembourg		2020-02-29	2020-04-19	US	Oregon	2020-03-01	2020-05-14
Malta		2020-03-07	2020-05-02	US	Pennsylvania	2020-03-07	2020-05-24
Nepal		2020-01-29	2020-05-24	US	Rhode Island	2020-03-03	2020-05-09
New Zealand		2020-03-02	2020-04-21	US	South Carolina	2020-03-09	2020-04-19
North Macedonia		2020-03-18	2020-04-21	US	South Dakota	2020-03-11	2020-05-24
Pakistan		2020-03-12	2020-05-08	US	Tennessee	2020-03-08	2020-04-27
Panama		2020-03-10	2020-05-12	US	Texas	2020-03-06	2020-05-24
Paraguay		2020-03-10	2020-05-03	US	Utah	2020-03-10	2020-05-24
Peru		2020-03-06	2020-05-24	US	Virginia	2020-03-09	2020-05-14
Portugal		2020-03-02	2020-05-01	US	Vermont	2020-03-10	2020-04-24
Romania		2020-03-13	2020-05-24	US	Washington	2020-02-07	2020-05-02
Russia		2020-03-07	2020-05-24	US	Wisconsin	2020-03-06	2020-04-27
Saudi Arabia		2020-03-02	2020-04-28	US	West Virginia	2020-03-11	2020-05-04
Senegal		2020-03-03	2020-05-24	US	Wyoming	2020-03-12	2020-05-12
Slovakia		2020-03-06	2020-04-13				

We test the effect of six different implemented measures on the containment of the SARS-CoV-2 virus: namely, school closures, shut-downs of non-essential businesses, mass gathering bans, travel restrictions in and out of risk areas, national border closures and/or complete entry bans, and nationwide curfews. The country and state governments and local health authorities have provided the type and timing of implemented measures. Most countries implement more than one measure simultaneously, so we also analyze combinations of the measures mentioned above and include the five most commonly implemented combinations and even one combination that consists of all other combinations. A summary of the key descriptives, the combinations of measures, and control variables can be found in Table 2.

**Table 2:** Descriptive statistics.

Variable	Description	Mean	Std. Dev.	Min	Max
*Dependent Variable*					
Confirmed cases	Cumulated number of confirmed cases over time	11810.29	35433.12	1	345,813
*Individual Measures*					
Risk area travel ban	Dummy variable indicating if a travel ban in and out of risk areas is implemented.	0.932	0.252	0	1
School closure	Dummy variable indicating if schools are closed.	0.849	0.358	0	1
Gathering ban	Dummy variable indicating if mass gatherings of more than 100 persons are banned.	0.831	0.374	0	1
Border closure	Dummy variable indicating if borders are closed or entry bans for foreigners are active.	0.785	0.411	0	1
Business shut-down	Dummy variable indicating if non-essential businesses are closed. Essential businesses include grocery stores, pharmacies, gas stations, and banks.	0.622	0.485	0	1
National curfew	Dummy variable indicating if a national curfew is implemented (includes only 24h-curfews).	0.114	0.318	0	1
*Combinations*					
No measure	Dummy variable indicating if no measure is active.	0.042	0.201	0	1
Travel ban only	Dummy variable indicating if only a travel ban in and out of risk areas is implemented.	0.061	0.241	0	1
Travel & school & gathering	Dummy variable indicating if a travel ban is active, schools are closed, and mass gatherings of more than 100 persons are banned.	0.033	0.179	0	1
Travel & school & gathering & border	Dummy variable indicating if a travel ban is active, schools are closed, mass gatherings of more than 100 persons are banned, and borders are closed, or entry bans for foreigners are operational.	0.155	0.362	0	1
Travel & school & gathering & border & business	Dummy variable indicating if a travel ban is active, schools are closed, mass gatherings of more than 100 persons are banned, borders are closed, or entry bans for foreigners are operational, and non-essential businesses are closed.	0.447	0.497	0	1
All & curfew	Dummy variable indicating if a travel ban is active, schools are closed, mass gatherings of more than 100 persons are banned, borders are closed, or entry bans for foreigners are operational, non-essential businesses are closed, and a national curfew is implemented.	0.113	0.316	0	1
Other combination	Dummy variable indicating if any combination of individual measures is active.	0.148	0.356	0	1
*Control variable*					
Population	The total population of the country or state (in 10,000).	1736.145	3242.126	3.372	21221.5
Number of daily tests	The number of tests on a given time t.	5181.891	16981.54	1	285,746
Detection & reporting index	Index for early detection and reporting for epidemics of potential international concern.	76.466	26.877	10.5	98.2
Rapid response index	Index for rapid response to and mitigation of the spread of an epidemic.	62.982	19.071	21.7	91.9
Health system index	Index for sufficient and robust health system to treat the sick and protect health workers.	56.527	19.886	7.5	73.8
Time	Observation period between the first confirmed case and either the first lifting of a measure or May 24.	40.809	24.120	1	121

Based on 6,941 observations.

Considering this diverse number of countries might be challenging as the number of cases can vary widely between countries and states over time due to differences in the cases’ definition, testing strategies, and reporting chain. In general, cases are categorized into three different levels: suspected, probable, and confirmed cases [12]. A suspected case shows clinical signs and symptoms of having COVID-19; a probable case has been in close contact with a positive case or a COVID-19-affected area; a confirmed case has laboratory confirmation of COVID-19 [12]. Our data include confirmed and probable cases that countries and states have stated. In addition, the definition of cases might change over time and results in abnormal spikes in the data. For example, France and some of the federal states of the United States of America add probable cases to their reporting in the observed period. France has included probable cases from nursing homes on April 12th [13], leading to an exponential increase of 26,849 cases on one single day.

Farther, the variation of total cases might be a result of different testing of COVID-19. The number of performed tests can be seen as an upper limit for the number of confirmed cases [14]. Therefore, a low number of tests can result in a low number of confirmed cases. The positive rate of tests can also vary by testing strategies. In the early phase of the pandemic, the resources of testing have been limited. Thus, the WHO recommends a prioritization that should focus on the early identification and protection of vulnerable patients and health care workers [15]. With growing testing capacity, the testing strategies shift to the prioritization of suspectable cases [15]. Still, this excludes the group of asymptomatic cases. Different studies (overview see [16]) show that asymptomatic cases can account for approximately 4% to 40% of the total cases. Concluding, a more comprehensive testing strategy can improve and increase case ascertainment [17].

In addition, the incubation period of a COVID-19 disease affects and probably delays the introduced measures to decelerate the proliferation of the SARS-CoV-2 virus. Thus, there is a difference between the actual reporting of a case and its infection (e.g., incubation period, plus time to seek testing, plus time to result and report). Lauer et al. [18] show that the incubation time can vary between 2 and 14 days, with an average of 5.1 days.

However, Lee et al. [19] have shown the superiority of predictive accuracy and data integration from multiple countries compared to individual country-based COVID-19 spread models. Following Lee et al. [19], we have built a comprehensive dataset to shed light on the impact of the different measures against the COVID-19 pandemic across countries based on improved models and declaration of variances. In addition, we have improved and extended our data description, focusing on the differences across countries and their data collection strategies. We have also included daily testing numbers across countries to account for testing bias that may affect the actual effects of the implemented restrictions. We have further integrated three indexes from the Global Health Security (GHS) to account for the reporting and investigation processes that can vary by country. The GHS Index is the comprehensive assessment and benchmarking of health security and related capabilities across 195 countries. The GHS Index is a Nuclear Threat Initiative (NTI) and Johns Hopkins Center for Health Security (JHU) project. In particular, we have included the following sub-indexes to our analyses:

1. Detection and Reporting: Early detection and reporting for epidemics of potential international concern
2. Rapid Response: Rapid response to and mitigation of the spread of an epidemic
3. Health System: Sufficient and robust health system to treat the sick and protect health workers

Following Lauer et al. [18], Li et al. [20], and Linton et al. [21], we have also included different incubation times to account for the difference between the report and the actual infection of COVID-19 cases. All studies have certain incubation times in common. For example:

- The minimum incubation time is approximately 2 days
- The average incubation time is approximately 5 days
- The 95 percentiles of the incubation time are approximately 11 days
- The maximum incubation time is approximately 15 days

We account for these incubation times and additionally include an incubation time of 8 days in our analyses. Therefore, we can account for the mean incubation of COVID-19 and control for possible additional time to seek testing, time to results, and/or time to report. We additionally control for the country sizes by including the population of each country and state.

## Method

We use the nonlinear mixed-effect model to analyze the relationship between the measures against the COVID-19 pandemic and the cumulated number of confirmed COVID-19 cases. The method is widely used for different epidemics such as influenza [22–24], hospital-acquired infections [25], HIV/AIDS [26], and SARS-CoV-2 [19, 27]. Nonlinear mixed-effect models outperform individual models by integrating information from different subjects to increase the predictive power for the individual [19]. It considers both random and fixed effects. Random effects allow the estimation of variance in the response variable within and among groups of variables [28]. We assume that observations of one country and state are closer to each other than those of other countries and states. Consequently, *country* and *state* are the grouping variables in our study. Fixed effects are represented by the predictor variables, which include the containment measures and the control variables.

**Fig 1:**
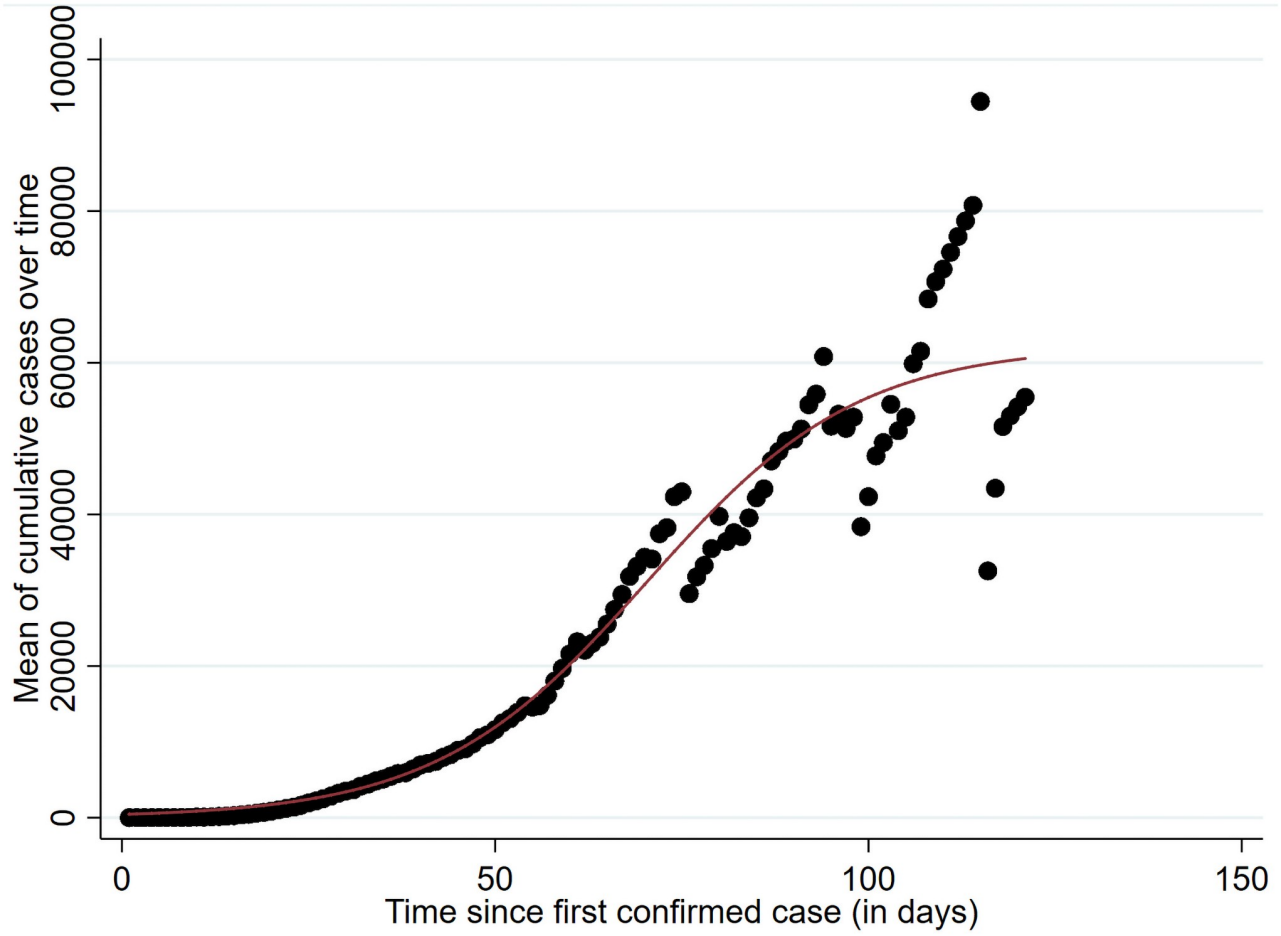
Logistic growth curve of the COVID-19 pandemic.

An additional advantage of the nonlinear mixed-effect model is the assumption of nonlinearity. Following Kamrujjaman et al. [29], Liang [30], and Roosa et al. [31], we also consider a logistic growth curve of the COVID-19 pandemic. The adjusted R^2^ of the logistic function is 98,05%, meaning that we have a nearly perfect overall fit of the represented logistic growth curve to our data (see Fig 1), which consists of three parameters, namely the upper asymptote, growth rate, and inflection point. In our analysis, the upper asymptote represents the final epidemic size. The growth rate represents the infection rate and, therefore, the speed of the spread. The inflection point represents the lag phase of the infection trajectory with the time point of daily cases’ peak [19].

As the logistic growth curve assumes that the pandemic follows the same logistic function in all observed countries and states, the expected cumulated number of confirmed cases over time will indeed differ to a certain degree, thus also affecting the expected inflection point and growth rate of the coronavirus outbreak. We account for these differences with random intercepts for the parameters (described in Eqs (2)–(4)). Following Bates and Pinheiro [32], we test the integration of random intercepts in the nonlinear mixed-effect model using the Akaike information criterion (AIC). We note that these information criteria suggest the inclusion of random intercepts for all three parameters: namely, the upper asymptote, growth rate, and inflection point. Thus, the model is represented by

$$Y_{ij}=\frac{\beta_{1j}}{\left(1+e^{-\beta_{2j}*\left(Time-\beta_{3j}\right)}\right)}+\varepsilon_{ij}$$ (1)

with

$$\beta_{1j}=\beta_{10}+\beta_{11}*measures+{ß}_{12}*controls+u_{1j}$$ (2)

$$\beta_{2j}=\beta_{20}+\beta_{21}*measures+{ß}_{22}*controls+u_{2j}$$ (3)

$$\beta_{3j}=\beta_{30}+\beta_{31}*measures+{ß}_{32}*controls+u_{3j}$$ (4)

Eq (1) specifies the logistic function for each country or state *i* at time *j*. *Y*_*ij*_ is the number of cumulated confirmed cases at time *j* for each country or state *i*. *β*_1*j*_ presents the upper asymptote (maximum size reached after an infinite growing time), *β*_2*j*_ shows the growth rate, and *β*_3*j*_ the time of the inflection point. *ε*_*ij*_ is the Gaussian error term of country or state *i* at time *j*.

Eqs (2)–(4) define the nonlinear mixed-effect model parameters at time *j*. *β*_10_, *β*_20_, and *β*_30_ are the fixed intercepts representing the mean estimates of the parameters. *u*_1*j*_, *u*_2*j*_, and *u*_3*j*_ represent the respective random intercepts for each country or state over time *j*. They account for the deviation of each country or state from the sample mean. The predictor variables are represented by the *measures* vector, including school closures, shut-downs of non-essential businesses, mass gathering bans, travel restrictions in and out of risk areas, national border closures and/or complete entry bans, and nationwide curfews. We also analyze the six most commonly implemented combinations of these individual measures. All analyses contain the control variables, which have been mean-centered [33, 34]. In addition, the control variables *population* and *daily tests* have been logarithmized.

## Results

Table 3 summarizes the estimations of the relationship between the measures and the confirmed coronavirus cases. Table 4 includes combinations of these measures to analyze the interactions between the measures and the cumulated confirmed COVID-19 cases. Nonlinear mixed-effect estimations are shown, representing the model outlined in Eqs (1)–(4). We estimate the parameters of the upper asymptote, growth rate, and time of the inflection point. The constants of the three parameters present the mean values without any implemented measures and at the mean of the control variables. Both tables include five models representing different reporting and incubation time lags.

**Table 3:** Nonlinear mixed-effect regression results of the individual measures.

Number of confirmed cases	Incubation	Incubation	Incubation	Incubation	Incubation
	2 days	5 days	8 days	11 days	15 days
**Upper asymptote**					
Risk area travel ban	-1,042.170 (2,369.086)	-194.506 (685.459)	123.214 (421.095)	889.533** (433.221)	554.648 (941.756)
School closure	-1,069.961 (1,542.001)	-743.277 (780.132)	-203.518 (369.106)	-127.165 (282.339)	-2,559.618*** (776.876)
Gathering ban	1,628.880 (2,626.511)	159.257 (901.092)	-541.432 (470.117)	-871.245** (400.771)	-2,004.077*** (751.200)
Border closure	324.431 (688.073)	656.658* (366.233)	642.198*** (235.505)	104.352 (230.547)	-1,494.998*** (361.433)
Business shut-down	-62.556 (331.834)	-8.769 (253.904)	14.403 (190.372)	37.005 (159.800)	-604.754*** (230.370)
National curfew	275.546 (577.580)	262.087 (516.108)	505.824 (431.389)	746.151 (478.385)	2,066.415*** (547.144)
Logarithm of population	21,026.824*** (3,627.763)	21,404.716*** (3,708.811)	21,744.965*** (3,811.321)	22,023.539*** (3,904.511)	21,500.910*** (4,025.339)
Logarithm of daily tests	-98.021** (42.405)	-65.582* (38.452)	-44.977 (33.814)	-22.118 (30.822)	7.826 (43.560)
Detection & reporting index	-241.479 (456.792)	-212.514 (467.037)	-174.375 (480.095)	-150.409 (492.146)	-145.132 (512.093)
Rapid response index	1,565.937** (667.813)	1,603.567** (683.045)	1,644.335** (702.025)	1,703.775** (719.090)	1,832.518** (747.701)
Health system index	-784.246 (787.584)	-856.416 (805.118)	-946.347 (827.521)	-1,032.826 (847.643)	-1,166.309 (882.670)
Constant	36,657.613*** (5,760.134)	37,296.887*** (5,658.121)	37,803.130*** (5,807.353)	38,380.807*** (5,949.906)	45,470.668*** (6,357.926))
**Growth rate**					
Risk area travel ban	-0.028*** (0.010)	-0.021*** (0.005)	-0.026*** (0.004)	-0.029*** (0.003)	-0.041*** (0.003)
School closure	0.015* (0.008)	0.018*** (0.005)	-0.003 (0.003)	-0.013*** (0.002)	-0.019*** (0.001)
Gathering ban	-0.010 (0.013)	-0.027*** (0.007)	-0.011*** (0.003)	-0.008*** (0.002)	-0.012*** (0.001)
Border closure	-0.055*** (0.007)	-0.056*** (0.004)	-0.052*** (0.003)	-0.039*** (0.002)	-0.021*** (0.001)
Business shut-down	-0.033*** (0.004)	-0.030*** (0.002)	-0.023*** (0.001)	-0.016*** (0.001)	-0.011*** (0.001)
National curfew	-0.012*** (0.003)	-0.009*** (0.002)	-0.005*** (0.001)	-0.004*** (0.001)	-0.003*** (0.001)
Logarithm of population	-0.010*** (0.004)	-0.009** (0.004)	-0.008** (0.003)	-0.008*** (0.003)	0.005** (0.002)
Logarithm of daily tests	0.002*** (0.000)	0.002*** (0.000)	0.002*** (0.000)	0.002*** (0.000)	0.001*** (0.000)
Detection & reporting index	-0.000 (0.000)	-0.000 (0.000)	-0.000 (0.000)	-0.000 (0.000)	0.000 (0.000)
Rapid response index	-0.002*** (0.001)	-0.001*** (0.001)	-0.001*** (0.0004)	-0.001*** (0.000)	-0.001* (0.000)
Health system index	0.001* (0.001)	0.001* (0.001)	0.001* (0.001)	0.001* (0.000)	0.001** (0.000)
Constant	0.214*** (0.017)	0.212*** (0.010)	0.207*** (0.007)	0.197*** (0.006)	0.175*** (0.005)
**Inflection Point**					
Risk area travel ban	1.176 (1.537)	0.668 (0.728)	1.519*** (0.418)	1.944*** (0.307)	2.180*** (0.246)
School closure	-2.998* (1.683)	-3.621*** (0.812)	-0.352 (0.307)	0.912*** (0.167)	1.357*** (0.115)
Gathering ban	-5.258 (4.051)	0.714 (1.427)	-1.689*** (0.644)	-1.374*** (0.299)	-0.027 (0.156)
Border closure	1.885*** (0.684)	2.924*** (0.397)	2.957*** (0.261)	1.914*** (0.161)	0.208** (0.095)
Business shut-down	0.641 (0.633)	1.471*** (0.326)	1.197*** (0.189)	0.849*** (0.124)	0.758*** (0.085)
National curfew	-0.553** (0.228)	-0.848*** (0.164)	-0.780*** (0.136)	-0.591*** (0.126)	0.478*** (0.116)
Logarithm of population	7.087*** (1.301)	7.204*** (1.285)	7.382*** (1.268)	6.949*** (1.198)	2.332* (1.220)
Logarithm of daily tests	-0.073** (0.037)	-0.021 (0.033)	-0.005 (0.029)	0.061** (0.025)	-0.060** (0.025)
Detection & reporting index	-0.031 (0.141)	-0.008 (0.140)	-0.021 (0.138)	-0.008 (0.136)	-0.023 (0.135)
Rapid response index	0.564*** (0.216)	0.545*** (0.208)	0.532** (0.207)	0.539*** (0.202)	0.652*** (0.203)
Health system index	-0.412* (0.247)	-0.414* (0.244)	-0.405* (0.242)	-0.452* (0.239)	-0.709*** (0.251)
Constant	55.760*** (4.573)	50.132*** (2.294)	48.680*** (1.877)	48.613*** (1.720)	55.970*** (1.838)
Observations	6,941	6,941	6,941	6,941	6,941
Number of countries/states	121	121	121	121	121

**Table 4:** Nonlinear mixed-effect regression results of the combinations of individual measures.

Number of confirmed cases	Incubation	Incubation	Incubation	Incubation	Incubation
	2 days	5 days	8 days	11 days	15 days
**Upper asymptote**					
Travel ban only	-172.445 (737.105)	84.326 (846.509)	3,215.602 (2,303.807)	-362.083 (534.269)	1,105.825* (612.972)
Travel & school & gathering	-689.856 (769.548)	-577.639 (533.291)	603.627 (697.065)	917.632* (478.995)	910.566** (386.303)
Travel & school & gathering & border	116.120 (507.022)	215.202 (425.861)	972.248 (647.673)	981.447** (446.496)	685.172** (347.333)
Travel & school & gathering & border & business	-127.002 (391.341)	-1.920 (332.866)	941.108 (641.193)	1,080.642** (447.188)	219.610 (340.702)
All & curfew	769.353 (671.282)	913.491 (635.023)	1,613.519* (823.718)	781.397 (842.119)	-3,031.869** (1,249.503)
Other combination	-45.533 (708.319)	74.010 (493.255)	1,017.596 (669.223)	1,237.000*** (449.209)	800.549** (347.838)
Logarithm of population	21,569.800*** (3,633.242)	21,277.077*** (3,692.174)	21,575.809*** (3,787.008)	21,832.108*** (3,869.800)	22,277.647*** (4,004.763)
Logarithm of daily tests	-94.255** (42.233)	-63.514 (39.207)	-43.156 (37.221)	-30.421 (33.928)	-44.103 (34.168)
Detection & reporting index	-281.663 (453.936)	-232.347 (465.230)	-208.670 (478.189)	-194.511 (490.306)	-174.566 (509.144)
Rapid response index	1,605.848** (664.017)	1,603.947** (679.811)	1,673.248** (698.167)	1,720.461** (715.397)	1,758.883** (742.823)
Health system index	-799.056 (781.596)	-843.626 (802.443)	-937.949 (824.894)	-1,018.290 (844.599)	-1,096.576 (877.763)
Constant	37,005.182*** (5,517.968)	37,163.159*** (5,631.949)	37,005.292*** (5,809.224)	37,729.696*** (5,930.052)	39,567.349*** (6,148.860)
**Growth rate**					
Travel ban only	0.001 (0.083)	0.000 (0.048)	-0.020 (0.014)	0.085*** (0.014)	-0.008*** (0.003)
Travel & school & gathering	0.003 (0.032)	0.013 (0.017)	-0.024*** (0.008)	-0.045*** (0.004)	-0.059*** (0.002)
Travel & school & gathering & border	-0.111*** (0.023)	-0.103*** (0.011)	-0.093*** (0.006)	-0.083*** (0.003)	-0.071*** (0.002)
Travel & school & gathering & border & business	-0.144*** (0.023)	-0.132*** (0.011)	-0.117*** (0.006)	-0.100*** (0.003)	-0.085*** (0.002)
All & curfew	-0.175*** (0.024)	-0.164*** (0.012)	-0.144*** (0.006)	-0.125*** (0.004)	-0.107*** (0.002)
Other combination	-0.080*** (0.022)	-0.072*** (0.011)	-0.066*** (0.006)	-0.059*** (0.003)	-0.054*** (0.002)
Logarithm of population	-0.009** (0.004)	-0.007** (0.003)	-0.005* (0.003)	-0.004 (0.003)	-0.002 (0.003)
Logarithm of daily tests	0.001*** (0.000)	0.002*** (0.000)	0.001*** (0.000)	0.002*** (0.000)	0.001*** (0.000)
Detection & reporting index	-0.000 (0.000)	-0.000 (0.000)	-0.000 (0.000)	-0.000 (0.000)	-0.000 (0.000)
Rapid response index	-0.002*** (0.001)	-0.002*** (0.000)	-0.001*** (0.000)	-0.001*** (0.000)	-0.001** (0.000)
Health system index	0.001 (0.001)	0.001 (0.001)	0.001 (0.001)	0.001 (0.000)	0.001 (0.001)
Constant	0.249*** (0.023)	0.231*** (0.012)	0.211*** (0.007)	0.189*** (0.005)	0.170*** (0.004)
**Inflection Point**					
Travel ban only	4.153 (7.756)	4.942 (5.022)	6.860*** (1.904)	-2.702*** (0.767)	1.924*** (0.347)
Travel & school & gathering	2.006 (3.772)	0.379 (1.746)	2.528*** (0.896)	3.737*** (0.501)	4.529*** (0.277)
Travel & school & gathering & border	9.291*** (3.531)	7.789*** (1.626)	6.841*** (0.829)	5.472*** (0.462)	4.079*** (0.259)
Travel & school & gathering & border & business	9.196*** (3.541)	8.333*** (1.636)	7.888*** (0.840)	6.499*** (0.472)	5.034*** (0.265)
All & curfew	14.530*** (3.575)	13.120*** (1.664)	11.403*** (0.858)	9.340*** (0.486)	7.460*** (0.278)
Other combination	7.805** (3.529)	6.878*** (1.624)	6.420*** (0.825)	5.524*** (0.456)	4.882*** (0.250)
Logarithm of population	7.656*** (1.361)	7.166*** (1.354)	6.314*** (1.187)	5.827*** (1.170)	5.659*** (1.189)
Logarithm of daily tests	-0.102*** (0.037)	-0.026 (0.034)	-0.007 (0.030)	0.043* (0.026)	-0.113*** (0.025)
Detection & reporting index	-0.065 (0.144)	-0.040 (0.143)	-0.062 (0.138)	-0.070 (0.139)	-0.065 (0.134)
Rapid response index	0.577** (0.216)	0.550** (0.216)	0.556*** (0.210)	0.552*** (0.208)	0.596*** (0.203)
Health system index	0.610*** (0.225)	0.551** (0.217)	0.558*** (0.210)	-0.435* (0.247)	0.628*** (0.202)
Constant	41.658*** (3.928)	43.280*** (2.380)	44.758*** (1.901)	46.832*** (1.780)	49.983*** (1.711)
Observations	6,941	6,941	6,941	6,941	6,941
Number of countries/states	121	121	121	121	121

The individual measures and combinations’ delayed effect mainly follows a quadratic relationship with a time lag maximum of eight to eleven days for the upper limit, a decreasing linear relationship for the growth rate, and mixed results between the estimation of individual measure and combinations for the inflection point. While the time delay of the pandemic primarily increases for the individual measures over the model specifications, the combinations show a decreasing relationship. The results of the constants and controls are broadly consistent, indicating robust findings across model specifications. However, some important distinctions can be made, most notably between the estimations that do and do not include the various combinations of measures.

Table 3 shows the nonlinear mixed-effect regression results of the individual measures. Following Lauer et al. [18], we focus on presenting our results on the five-day incubation time.

On average, we have a total number of 37,297 cumulated confirmed cases, with an average growth rate of 21.2%. The time of inflection point is around 50 days after the first confirmed case of COVID-19. We find no statistically significant results pointing towards the impact of the individual measures on the upper asymptote, except for border closure, which correlates positively with the upper limit with around 657 cases. This relationship changes for a time delay of 15 days; here, we find a significant negative relationship between the school closure, ban of gathering, border closure, business shut-down, and the upper limit.

The individual measures indeed have a statistically significant impact on the growth rate. For example, a risk area travel ban is associated with a decrease in the growth rate by 2.1 percentage points, while the ban of gatherings leads to a reduction of 2.7 percentage points. Closing borders and businesses are also negatively associated with daily growth rates of infection: closing of borders is found to decrease the growth rate by 5.6 percentage points. In comparison, closing businesses is related to a decrease of around 3 percentage points. National curfews lead to an average decrease in growth rate by 0.9 percentage points. A significant negative relationship between school closure and the growth rate can be found for an eight- and eleven-time delay.

In addition, closing borders and businesses and closing schools have a statistically significant impact on the time of the inflection point: Closing borders and business is associated with a postponement of the inflection point by three and one days, respectively, while school closing leads to a preponement of approximately three to four days.

Table 4 shows the nonlinear mixed-effect regression results of the combinations of individual measures. The signs and relative magnitudes of the constants and control variables suggest that the estimations of the individual measures are consistent with the analyses of the combinations of measures. We also find no statistical significance of the combinations of measures on the upper asymptote. Though, a time delay of eleven to fifteen days shows a positive relationship between the combinations and cumulated number of confirmed cases.

However, the combinations of measures are negatively associated with the growth rate. This significant effect increases with the number of individual measures included in the combinations. While the combination of risk area travel bans, school closures, bans of gatherings, and border closure leads to a decrease in the growth rate by 10.3 percentage points, the combination of all individual measures (including a national curfew) reduces the growth rate of confirmed COVID-19 cases by 16.4 percentage points. This same combination is associated with a postponement of the inflection point by 13 days. In addition, the combinations of "risk area travel bans, school closures, bans of gatherings, closed borders" and "risk area travels bans, school closures, bans of gatherings, closed borders, business closures" postpone the inflection point by 7.7 days and 8.3 days, respectively.

The results of control variables are broadly consistent within the models and specifications. The population size increases the upper asymptote and delays the inflection point; a one percent deviation from the mean increases the upper asymptote by 21,277 confirmed cases and delays the inflection point by seven days. The number of daily tests shows no economically significant results. While the rapid response index has a significant positive relationship with the upper limit, the health system index shows the contrary effect. Both correlate with a delay of the inflection point. An increase of 10 points in the indexes is associated with a delay of around five days.

**Fig 2:**
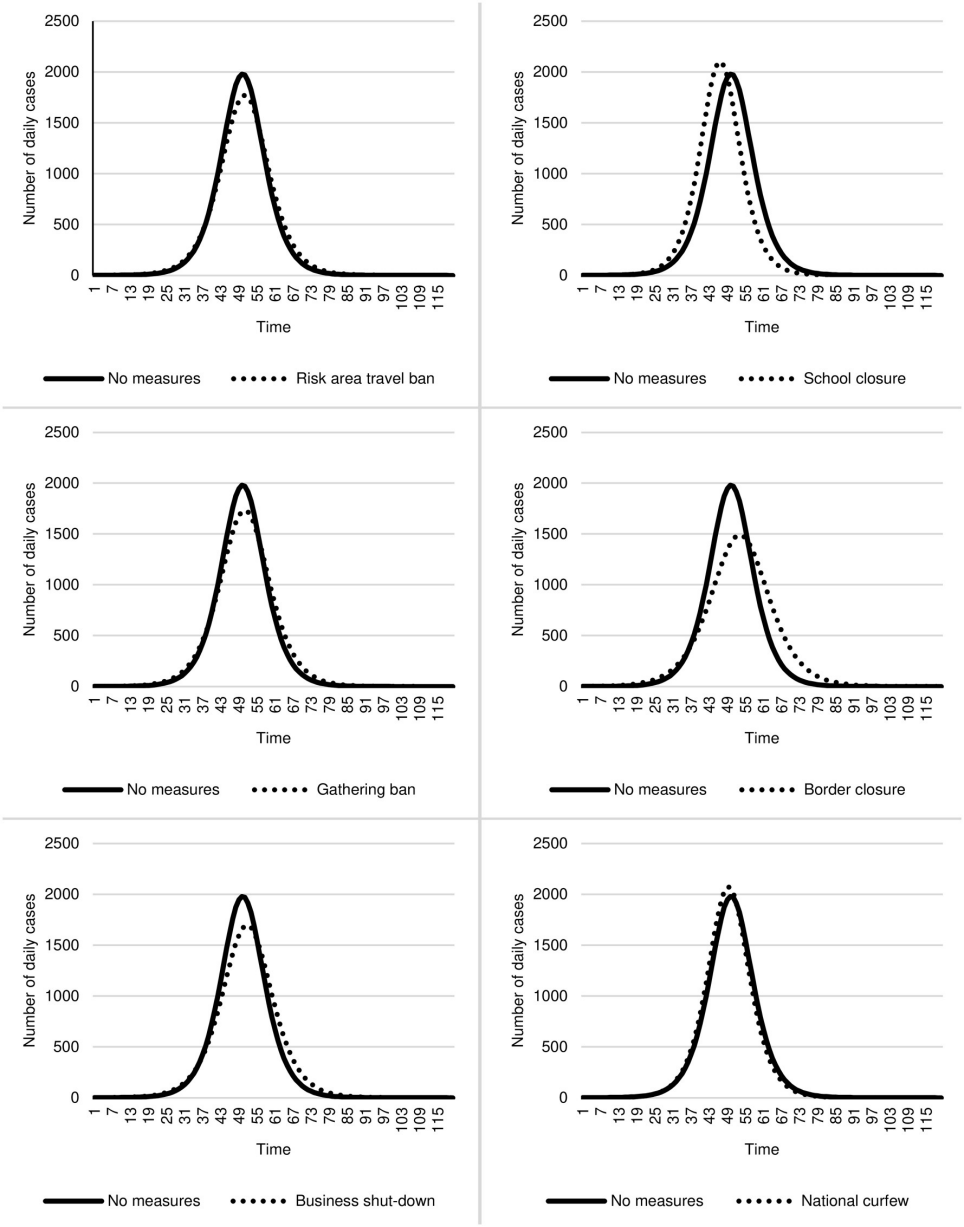
Flatten the curve—The effect of individual measures on the containment of the COVID-19 pandemic (incubation of 5 days).

**Fig 3:**
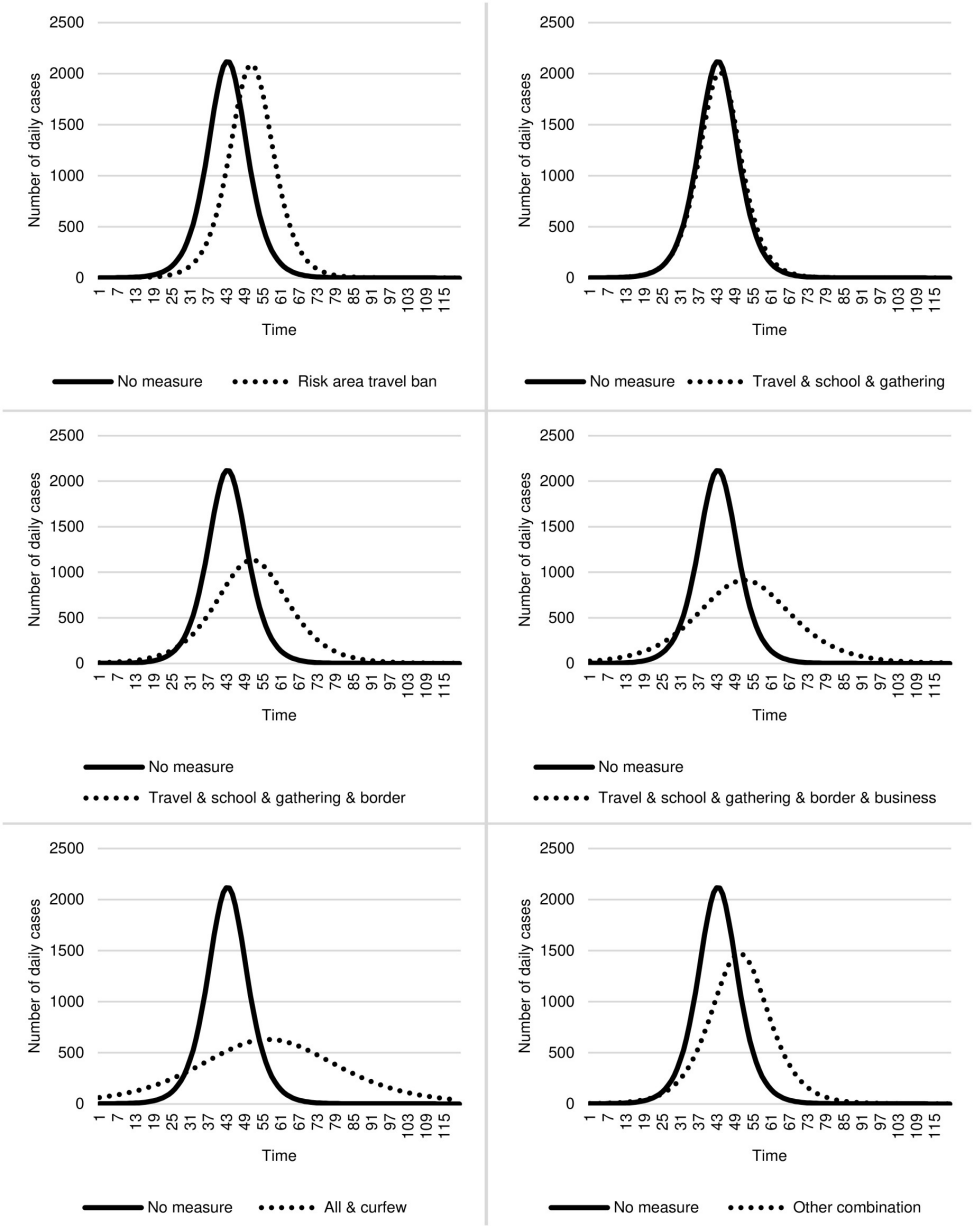
Flatten the curve—The effect of combinations of measures on the containment of the COVID-19 pandemic (incubation of 5 days).

The reduction of the growth rate and postponement of the peak can facilitate the containment of the COVID-19 pandemic and, thus, ease any overload of healthcare systems’ capacity. Following the Centers for Disease Control and Prevention [35], we examine the benefits of the containment measures against the COVID-19 pandemic and show the "flatten the curve" graphs for a time delay of five days in Fig 2 (for individual measures) and Fig 3 (for combinations of measures). The results show that individual measures can reduce daily cases’ peak by up to 490 cases on average (see Fig 2). For example, implementing a gathering ban reduces the daily cases’ peaks by 234 cases while border closure reduces 490 cases. This effect is even more substantial when measures are combined. Here, the peak decreases by up to 1,205 cases on average (see Fig 3). While implementing a risk area travel ban only delays daily cases’ peak by five days, the combination of measures delays 13 days.

In summary, it can be stated that all individual measures significantly decrease the daily growth rates of coronavirus cases. Moreover, the various combinations of these measures and the combinations of all measures significantly decrease the growth rates of confirmed cases, outweighing the individual measures’ growth rates. Particular measures not only decrease but also postpone the peak of infection of new COVID-19 cases.

## Concluding remarks

Governments worldwide are searching for policies to respond to the ongoing outbreak of the COVID-19 pandemic caused by the severe acute respiratory syndrome, coronavirus 2 (SARS-CoV-2) [36]. These policy measures aim to advance the containment of the spread of the COVID-19 pandemic by reducing social contact among individuals within or between populations [37]. Among others, these social distancing policies include restricting travel, closing schools, and restricting populations to their homes through a complete lockdown [38]. Using data from six countries (China, South Korea, Italy, Iran, France, and the United States), Hsiang et al. [39] have simulated that the combined effect of policies within each country prevented or delayed the growth rate of infections by approximately 61 million confirmed cases and thus, significantly slowed the outbreak of the pandemic in these countries. However, the actual effects of these policies and measures on infection rates worldwide during this ongoing coronavirus pandemic need to be investigated.

Using data from 68 countries, Puerto Rico and the 50 federal states of the United States of America, four federal states of Australia, and eight federal states of Canada, covering 6,941 daily observations between January 22 and May 24, 2020, we show that federal policies have been effective in containing the spread of the COVID-19 pandemic. We show that all individual measures (i.e., school closures, shut-downs of non-essential businesses, mass gathering bans, travel restrictions in and out of risk areas, national border closures and/or complete entry bans, and nationwide curfews) significantly decrease the growth rate of confirmed cases. However, combinations of these measures outweigh the effects of the individual measures. Thus, combinations of policies are the most suitable means for significantly slowing both the regional and the global outbreak of the coronavirus.

Despite the range of unique results and recommendations outlined by this study, our work suffers from several limitations. One concern pertains to the data itself. Countries and states report confirmed COVID-19 cases that have been formerly tested and identified as corona-positive. However, these reported numbers do not represent the "real" numbers of infections and only show the cases that tests have positively confirmed. Thus, asymptomatic infected people and patients having COVID-19 but not being sent to testing are not part of the statistics of the confirmed cases. Additional studies [17, 40–42] have used machine learning techniques to simulate the "real" number of COVID-19 cases. All models have produced valid results to conclude that the number of "real" infections far outnumber the reported confirmed cases. Nevertheless, they all follow the same growth curve of the COVID-19 pandemic and confirm the growth rate and inflection point but differ in the upper limit.

The number of hospitalized cases in intensive care units instead of cumulative confirmed cases could also be used as a substitute measure. This variable could represent an adequate robustness test as the COVID-19 intensive care unit patients are related to the number of cases but independent of the number of tested people.

Additionally, we observe the proliferation of the SARS-CoV-2 virus until the end of May 2020. As the COVID-19 pandemic is still ongoing and prevalent across countries, future studies could extend the time frame and include periods before introducing vaccinations. Our analysis also assumes that the implemented measures have the same impact in observed countries and states. Several studies have shed light on the differences in compliance with measures dependent on the stage of the COVID-19 pandemic [43], the trust in the health care system [44], or country differences [45]. Furthermore, we "only" observe measures implemented by the government and law institutions. Still, social norms, cultural differences, and behavioral recommendations can also be effective interventions for containing the spread of the COVID-19 pandemic. Consequently, future studies could focus on experiments and surveys that explain the compliance of non-law policies and non-pharmaceutical interventions in the population.

In summary, non-pharmaceutical interventions can be an effective measure for containing the spread of the COVID-19 pandemic. Therefore, health offices can apply measures such as social distancing and travel restrictions to decelerate the proliferation of the SARS-CoV-2 virus and, thus, "flatten the curve".
